# Evaluation of anti-inflammatory activity of compounds isolated from the rhizome of *Ophiopogon japonicas*

**DOI:** 10.1186/s12906-016-1539-5

**Published:** 2017-01-05

**Authors:** Jing-Wen Zhao, Ding-Sheng Chen, Chang-Sheng Deng, Qi Wang, Wei Zhu, Li Lin

**Affiliations:** 1 The Second Institute of Clinical Medicine, Guangzhou University of Chinese Medicine, Guangzhou, 510405 Guangdong China; 2 Guangdong Food and Drug Vocational College, Guangzhou, 510520 Guangdong China; 3 Artepharm Company Limited, Guangzhou, 510410 Guangdong China

**Keywords:** Ophiopogon japonicus, Homoisoflavonoids, Anti-inflammatory, Macrophages, MAPKs

## Abstract

**Background:**

*Ophiopogon japonicas* (L.f) Ker-Gawl has been used as a traditional Chinese medicine to cure acute and chronic inflammation and cardiovascular diseases including thrombotic diseases for thousands of years. Previous phytochemical studies showed that *O. japonicus* contained compounds with anti-inflammatory activity. The aim of this study was to identify and isolate compounds with anti-inflammatory activity from the rhizome of *O. japonicas*.

**Methods:**

Compounds were isolated by various column chromatography and their structures were identified in terms of nuclear magnetic resonance spectrum (NMR) and mass spectrum (MS). To measure the anti-inflammatory effects of thirteen compounds in LPS-induced RAW 264.7 macrophage cells, we used the following methods: cell viability assay, nitric oxide assay, enzyme-linked immunosorbent assay, quantitative real-time PCR analysis and western blotting analysis.

**Results:**

One new and twelve known compounds (mainly homoisoflavonoids) were extracted from *O. japonicas*, in which 4′-*O*-Demethylophiopogonanone E (10) was considered as a new compound, additionally, compounds 4-*O*-(2-Hydroxy-1- hydroxymethylethyl)-dihydroconiferyl alcohol (2) and 5,7-dihydroxy-6-methyl-3-(2′, 4′-dihydroxybenzyl) chroman-4-one (12) were isolated from the rhizome of *O. japonicas* for the first time. The isolated compounds Oleic acid (3), Palmitic acid (4), desmethylisoophiopogonone B [5,7-dihydroxy-3-(4′-hydroxybenzyl)-8- methyl- chromone] (5), 5,7-dihydroxy-6-methyl-3-(4′-hydroxybenzyl) chromone (7) and 10 significantly suppressed the production of NO in LPS-induced RAW 264.7 cells. Especially compound 10 showed the strongest effect against the production of the pro-inflammatory cytokine IL-1β and IL-6 with the IC_50_ value of 32.5 ± 3.5 μg/mL and 13.4 ± 2.3 μg/mL, respectively. Further analysis elucidated that the anti-inflammatory activity of compound 10 might be exerted through inhibiting the phosphorylation of ERK1/2 and JNK in MAPK signaling pathways to decrease NO and pro-inflammatory cytokines production.

**Conclusions:**

Our results indicated that 4′-*O*-Demethylophiopogonanone E can be considered as a potential source of therapeutic medicine for inflammatory diseases.

## Background

Inflammation is a biological response of tissue in attempting self-protection against harmful stimuli, caused by a mechanical or biological agent or by an aberrant autoimmune response [1]. Macrophages play a vital role in inflammatory response in the initiation, maintenance and resolution of inflammation [2]. In macrophages, lipopolysaccharide (LPS), a well-known endotoxin, induces the release of numerous pro-inflammatory mediators such as inducible nitric oxide synthase (iNOS) and inflammatory cytokines, including interleukin-1β (IL-1β) and interleukin-6 (IL-6), which play a major role in the pathogenesis of various inflammatory disorders and serve as significant biomarkers for the assessment of the inflammatory process [3–6]. iNOS is a protein whose expression is regulated by activation of NF-κB, which contribute to the production of NO [7, 8]. Furthermore, mitogen-activated protein kinases (MAPKs), including extracellular signal-regulated kinases (ERK1/2), c-Jun NH_2_-terminal kinases (JNK) and p38, play important roles in regulation of the inflammatory response by mediators. The signaling pathways of MAPKs can lead to the activation of NF-κB and induce expression of pro-inflammatory genes, including IL-1β, IL-6 and iNOS [9–11].

The plant *Ophiopogon japonicus* (L.f) Ker-Gawl is widely distributed in Southeast Asia, especially in most areas of China. Its rhizome is the primary medical portion and has been used as a traditional Chinese medicine to treat the inflammatory diseases for thousands of years [12]. *O. japonicus* is an important nourishing-yin drug in the traditional Chinese herbs with various bioactivities, including anti-inflammation, anti-cardiovascular diseases, anti-tumor, anti-aging, immunoregulation [13]. Previous phytochemical studies showed that the rhizome of *O. japonicas* contained homoisoflavonoids, saponins, amides, monoterpene glycosides, and so on [14].

The study of the biological functions of *O. japonicas* has been limited largely to demonstration of antioxidant activities *in vitro*. Few studies have been performed to explore the relationship between the bioactive constituents of *O. japonicas* and their anti-inflammatory properties. Moreover, the molecular mechanisms underlying anti-inflammatory activities of *O. japonicus* remain unclear in the literature. In this report, we describe the isolation and identification of the compounds extracted from *O. japonicus*, followed by an evaluation of the anti-inflammatory activities of these compounds. This work was aimed to identify the chemical components with anti-inflammatory activity from *O. japonicus* and to demonstrate their targeted pathway by the use of a bioactivity guided experimental design.

## Methods

### Plant materials

The radix of *O. japonicus* were purchased from Kangmei Pharmaceutical Co. Ltd., Guangdong, China and were authenticated by Ph. D. Zhi-Hai Huang, department of the Second Institute of Clinical Medicine, Guangzhou University of Chinese medicine, Guangzhou, China. The voucher specimens are deposited at the Guangdong Provincial Academy of Chinese Medical Sciences for future reference, and the voucher specimen number is 110811881.

### Instruments

1D-and 2D-NMR spectra data were obtained on a Bruker Avance 300 and 500 NMR spectrometer, with TMS as an internal standard. Electrospray ionization mass spectra (ESI-MS) were measured on a Thermo Scientific Finnigan LTQ mass spectrometer, and Preparative HPLC was conducted using a Waters 2545 Binary gradient module instrument with 2998 Photodiode Array Detector. Column chromatography (CC) separations were performed with D101 resin column (Beijing greenherbs Co. Ltd., China), silica gel (100–200 mesh, Qingdao Haiyang Chemical Co. Ltd., China), Sephadex LH-20 (Pharmcia Biotech AB, Uppsala, Sweden). TLC was carried out on glass precoated silica gel GF_254_ plates (Yantai Chemical Industrial Institute, China) and spots were visualized under UV light ((k = 254 nm or 366 nm) or by spraying with 10% (v/v) sulfuric acid in ethanol followed by heating to 105 °C. Real-time PCR (Rrism 7500, Applied Biosystems), Nano Drop 2000C Spectrophotometer (Thermo Scientific) and Chemidoc imaging system (BIO-RAD) were for the cell assay.

### Extraction and isolation

**Fig. 1:**
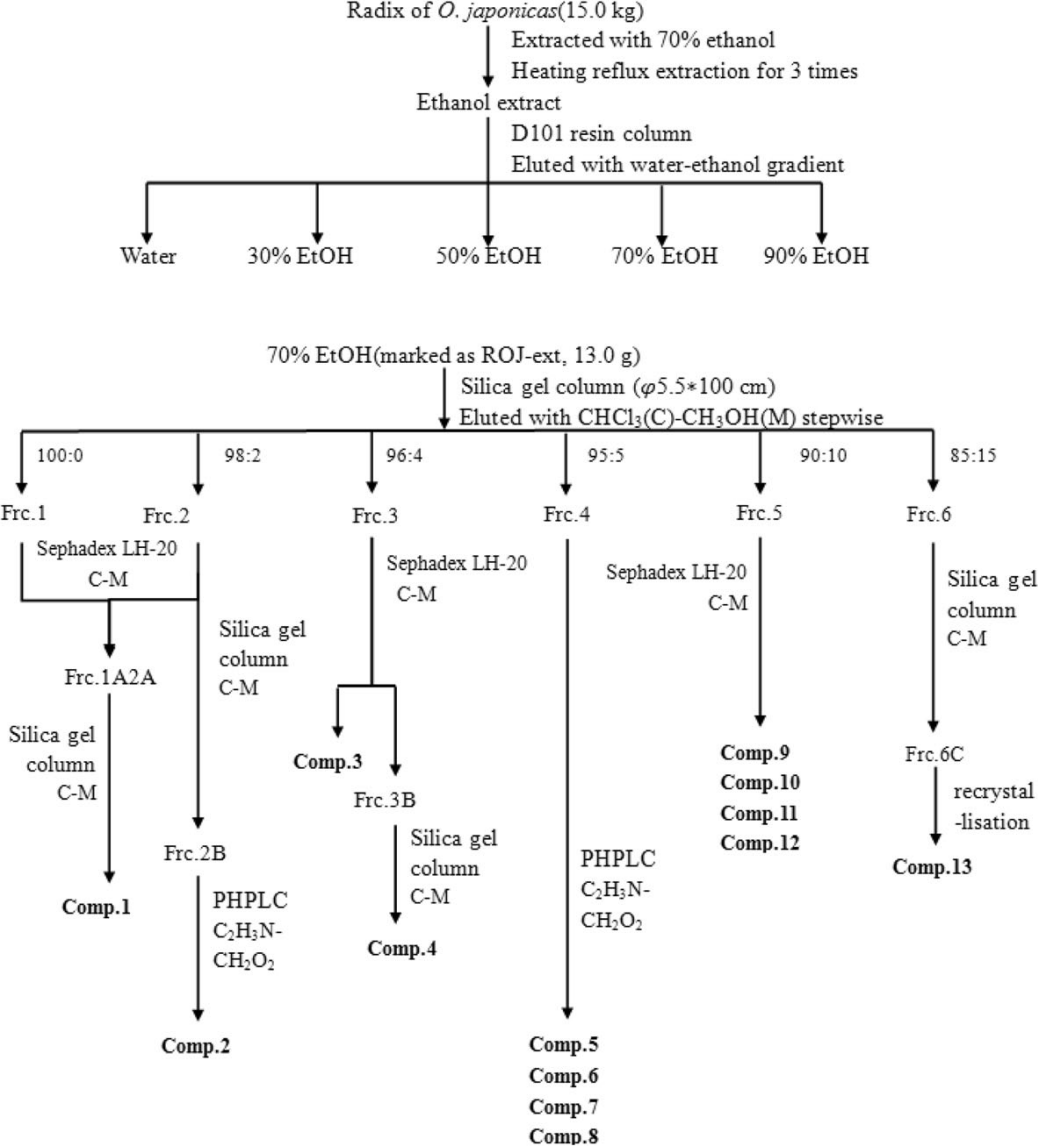
Isolation processes of compounds 1–13

The extraction and isolation of the compounds was shown in Fig. 1. The rhizome of *O. japonicus* (15.0 Kg) was exhaustively extracted through refluxing three times with 70% ethanol (15 × 3 L) for 2 h. Then the aqueous alcohol solution was evaporated under reduced pressure at 55 °C. The concentrated liquor was mixed with four times the volume of 95% ethanol solution, overnight at 4 °C, then filtered and concentrated under vacuum to yield a viscous residue. The residue was suspended in water, and then was subjected to a D101 resin column chromatography and eluted successively with water, 30%, 50%, 70% and 90% ethanol. The 70% ethanol elution was collected and concentrated under vacuum to yield the extraction (marked as ROJ-ext, 13.0 g). The ROJ-ext was subjected to silica gel column eluted with CHCl_3_-CH_3_OH (100:0 → 85:15) to obtain six subfractions (Frc.1-6). Frc.1 and Frc.2 was applied to Sephadex LH-20 with CHCl_3_-CH_3_OH (1:1) as an eluant to provide Frc.1 A2A, and then were further purified by silica gel column eluted with CHCl_3_-CH_3_OH (100:1) to afford compound 1 (15.7 mg). Frc.2 B was applied to PHPLC with C_2_H_3_N-CH_2_O_2_ (35:65) as an eluant to provide (compound 2, 10.4 mg). Frc.3 was applied to Sephadex LH-20 with CHCl_3_-CH_3_OH (1:1) as an eluant to provide Frc.3 A (compound 3, 19.4 mg) and Frc.3 B, the Frc.3 B was purified by silica gel column eluted with CHCl_3_-CH_3_OH (100:1 → 100:2) to afford compound 4 (6.2 mg). Frc.4 was applied to PHPLC with C_2_H_3_N-CH_2_O_2_ (35:65) as an eluant to provide compound 5 (10.7 mg), compound 6 (9.9 mg), compound 7 (10.2 mg) and compound 8 (10.9 mg). Frc.5 was applied to Sephadex LH-20 with CHCl_3_-CH_3_OH (1:1) as an eluant to provide compound 9 (9.7 mg), compound 10 (9.5 mg), compound 11 (6.2 mg) and compound 12 (8.1 mg). Frc.6 was applied to silica gel column eluted with CHCl_3_-CH_3_OH (100:5 → 100:7 → 100:9) and re-crystallisation to provide compound 13 (7.3 mg).

### Cell culture

The mouse RAW264.7 macrophage cell line was purchased from the cell bank of the Chinese Academy of Sciences (CAS, Shanghai, China). Cells were maintained at 37 °C in a humidified atmosphere containing 5% CO_2_ in Dulbecco′s Modified Eagle Medium (DMEM, Sigma, St. Louis, Mo, USA) supplemented with 10% heat-inactivated fetal bovine serum (FBS, Sigma).

### Cell viability assay

The cytotoxicity of the isolated compounds toward RAW264.7 macrophage cells were evaluated by a conventional MTT assay as reported previously [15]. RAW 264.7 cells (1.0 × 10^5^cells/well) were given a volume of 100 μl to 96-well plates and incubated for 24 h and then treated with or without different concentrations of compounds (1, 5, 25, 50 and 100 μg/mL). Dimethyl sulphoxide (DMSO) was used to dissolve the dried samples, and its final concentration of DMSO in the culture medium was maintained at less than 0.1% (v/v). The culture plates were kept at 37 °C with 5%CO_2_ and the assay for each concentration of extracts were performed in triplicates. After additional 24 h incubation, the medium was discarded, and then new medium without PBS was added, and the cells were incubated for 4 h with a solution of 5 mg/mL MTT. The supernatant was removed carefully, and 200 μL DMSO was added to dissolve the formazan crystals. The plate was shaken for 10 mins, and the absorbance was recorded on a Thermo Scientific microplate spectrophotometer (Thermo Fisher Scientific Inc., Waltham, MA, USA) at the wavelength of 490 nm.

### Nitric oxide assay

The accumulation of nitrite, an indicator of NO production in the culture medium, was measured with the Griess reagent [16]. RAW264.7 cells were plated at 4 × 10^5^ cells/mL in 24 wells- culture plates. After 24 h incubation, cells were pre-incubated in medium with or without various concentrations of compounds for 2 h. The experimental groups were then stimulated with LPS (final concentration 1 μg/mL) at 37 °C for another 24 h, in while the dexamethasone (DXM) group at the concentration of 50 μg/mL was as a positive control. Subsequently, the supernatant was collected. Fifty microlitres of cell culture medium were mixed with 50 μL of Griess reagent (equal volumes of 1% sulphanilamide in 5% phosphoric acid and 0.1% N-1-naphtylethylenediamine dihydrochloride in distilled water), incubated at room temperature for 10 min, and then the absorbance at 540 nm was measured in a microplate reader. Nitrite concentrations in the supernatant were determined by comparison with a sodium nitrite standard curve.

### Enzyme-linked immunosorbent assay

The supernatant above-mentioned in the 24 wells-culture plates were collected. Pro-inflammatory cytokines (IL-1β and IL-6) levels in culture medium were determined using commercially available enzyme-linked immunosorbent assay (ELISA) kits (R&D Systems) according to the manufacturer’s instructions.

### Quantitative real-time pcr analysis

The cells were seeded in 6-well plates (1.0 × 10^6^ cells/well) and the groups were the same as above. The total RNA was extracted with Tripure reagent. The total RNA was stored at −80 °C until use. Then Transcriptor First Stand cDNA Synthesis Kit (Roche, USA) was used to reverse transcribe complementary DNA. Subsequently, quantitative real-time PCR was performed by ABI Prism7500 Sequence Detection System using SYBR Green Master Rox (Roche, USA) at the standard conditions. The nucleotide sequences of the primers used were as follows: IL-1β (forward, 5′-TGA AGG GCT GCT TCC AAA CCT TTG ACC-3′, reverse, 5′-TGT CCA TTG AGG TGG AGA GCT TTC AGC-3′), IL-6 (forward, 5′-TAC TCG GCA AAC CTA GTG CG-3′, reverse, 5′-GTG TCC CAA CAT TCA TAT TGT CAG T-3′), iNOS (forward, 5′- CGG CAA ACA TGA CTT CAG GC -3′, reverse, 5′- GCA CAT CAA AGC GGC CAT AG -3′), GAPDH (forward, 5′-TTT GTC AAG CTC ATT TCC TGG TAT G-3′, reverse, 5′-TGG GAT AGG GCC TCT CTT GC-3′). The results were expressed as the ratio of optical density to GAPDH. Relative expression levels were calculated using the 2^−△△Ct^ method [17]. All qRT-PCR assays were repeated at three times.

### Western blotting analysis

Western blotting was performed according to a standard method [18]. The cells were seeded in 6-well plates (1.0 × 10^6^ cells/well) and the groups were the same as above. Proteins were extracted from cells in the ice-cold RIPA lysis buffer (50 mM Tris–HCl, pH 7.4, 150 mM NaCl, 1% Nonidet P-40, 0.5% sodium deoxycholate, 0.1% SDS) containing with PMSF, and incubated with 40 min on the ice. After centrifugation at 12,000 rpm for 20 min, the supernatant was collected. The protein concentration was determined using the BCA kit (Sigma-Aldrich) according to the manufacturer’s instruction. The protein from each sample was boiled with loading buffer for 10 min, forty microgram of protein per lane was electrophoresed through 12% SDS-PAGE gel, and followed by transferring to a PVDF membranes, which were activated in methanol. The membrane was blocked with 5% skim milk for 1 h at room temperature, and then incubated with primary antibodies (Cell Signaling Technology Inc., Beverly, MA, USA) at 1:1000 (v/v) dilution in 5% BSA at 4 °C overnight, and washed three times with TBST buffer. The membrane was followed by incubation for 1 h at room temperature with horseradish peroxidase-conjugated anti-rabbit IgG secondary antibody (CST). Then the blots were washed with TBST buffer three times and visualized by an enhanced chemiluminescent (ECL) detection solution.

### Statistical analysis

Statistical comparisons were performed between the control and treated groups. The data obtained from three independent experiments were reported as mean values ± standard deviations. Statistical analysis was carried out using SPSS 19.0 software. The data were subjected to analysis of variance (ANOVA) for comparing three or more groups, a significant difference was assumed at a level of *p* < 0.05.

## Results and discussion

### Isolation and identification of the compounds

**Fig. 2:**
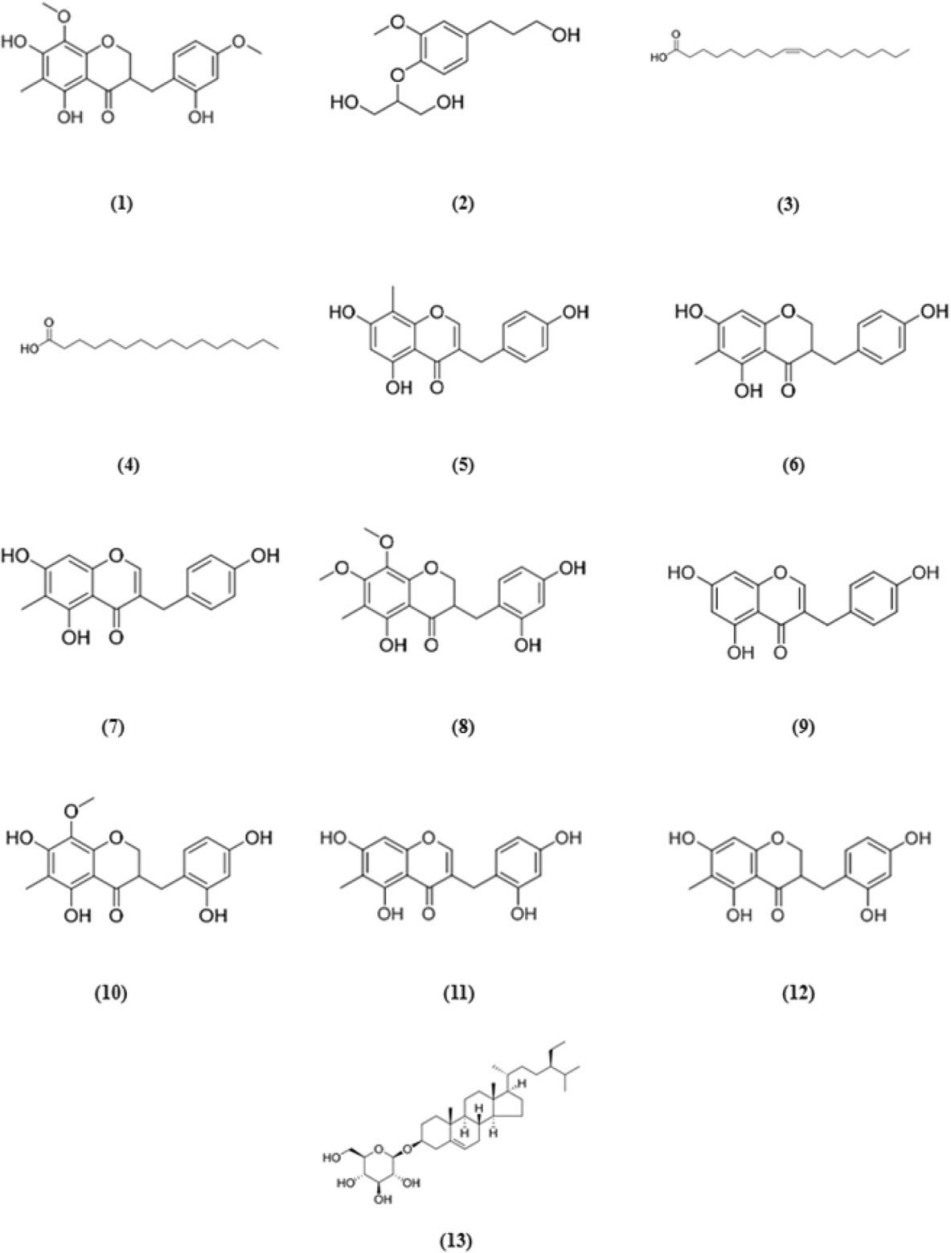
Chemical structures for compounds 1–13 isolated from ROJ-ext

The rhizome of *O. japonicus* was extracted with 70% ethanol, and the extracts were subjected to D101 resin column, silica gel column and Sephadex LH-20 for fractionation. Repeated column chromatography was performed for each fraction to yield 13 compounds. The structures of the isolated compounds were identified by comparing their ESI-MS, 1D- and 2D-NMR spectroscopic data with values reported in the literature, as Ophiopogonanone E (1); 4-*O*-(2-Hydroxy-1-hydroxymethylethyl)- dihydroconiferyl alcohol (2); Oleic acid (3); Palmitic acid (4); desmethylisoophiopogononeB [5,7-dihydroxy-3-(4′-hydroxybenzyl)-8-methylchromoe] (5); 5,7-dihydroxy-6-methyl-3-(4′-hydroxy-benzyl)-chroman-4-one (6); 5,7-dihydroxy −6-methyl-3-(4′-hydroxybenzyl) chromone (7); 3-(2,4-Dihydroxybenzyl)-5-hydroxyl- 7,8-dimethoxy-6-methylchroman-4-one (8); 5,7-dihydroxy-3-(4′-hydroxybenzyl) chromone (9); 4′-*O*-Demethylophiopogonanone E (10); Ophiopogonone D (11); 5,7- dihydroxy-6-methyl-3-(2′,4′-dihydroxybenzyl) chroman-4-one (12); Daucosterol (13) respectively. Among these compounds, compound 10 was considered as a new compound, additionally, compound 2 and 12 were isolated from the rhizome of *O. japonicus* for the first time. The spectral data of the 13 compounds were described in detail below and the structures were shown in Fig. 2.

*Ophiopogonanone E* (1). Yellow amorphous powder; Its positive-ion ESI-MS (*m*/*z*) displayed quasi-molecular ion peaks at 361 [M + H]^+^, 743 [2 M + Na]^+^, indicating a molecular weight of 360, molecular formula of C_19_H_20_O_7_. ^1^H-NMR (DMSO-*d*_*6*_, 500 MHz): δ 12.28 (1H, s, 5-OH), 6.95 (1H, dd, *J* = 8.3 Hz, H-6′), 6.40 (1H, d, *J* = 2.6 Hz, H-3′), 6.34 (1H, dd, *J* = 8.3, 2.6 Hz, H-5′), 4.28 (1H, dd, *J* = 11.4, 4.3 Hz, H-2a), 4.14 (1H, dd, *J* = 11.4, 7.4 Hz, H-2b), 3.67 (3H, s, 4′-OCH_3_), 3.63 (3H, s, 8-OCH_3_), 3.04 (1H, dd, *J* = 13.6, 5.1 Hz, H-9a), 2.95 (1H, m, H-3), 2.55 (1H, dd, *J* = 13.6, 9.6 Hz, H-9b), 1.90 (3H, s, 6-CH_3_). ^13^C-NMR (DMSO-*d*_*6*_, 125 MHz): δ 69.3 (C-2), 44.4 (C-3), 198.5 (C-4), 156.6 (C-5), 103.4 (C-6), 157.5 (C-7), 127.8 (C-8), 26.7 (C-9), 151.6 (C-10), 100.8 (C-11), 116.5 (C-1′), 156.3 (C-2′), 101.3 (C-3′), 159.0 (C-4′), 104.2 (C-5′), 131.3 (C-6′), 7.3 (6-CH_3_), 54.9 (4′-OCH_3_), 60.8 (8-OCH_3_). Based on the above results and compared the spectral data with literature [19], compound 1 was identified as Ophiopogonanone E.

*4*-*O*-(*2*-*Hydroxy*-*1*-*hydroxymethylethyl*)-*dihydroconiferyl alcohol* (2). White amorphous powder; Its positive-ion ESI-MS (*m*/*z*) displayed quasi-molecular ion peaks at 279 [M + Na]^+^, indicating a molecular weight of 256, molecular formula of C_13_H_20_O_5_. ^1^H-NMR (CD_3_OD, 300 MHz): δ 6.98 (1H, d, *J* = 8.2 Hz, H-5), 6.85 (1H, d, *J* = 2.0 Hz, H-2), 6.73 (1H,dd, *J* = 8.2, 2.0 Hz, H-6), 4.15 (1H, m, H-2′), 3.83 (3H, s, OCH_3_), 3.68-3.80 (4H, m, H-1′, H-3′), 3.55 (2H, t, *J* = 6.5 Hz, H-9), 2.62 (2H, t, *J* = 7.7 Hz, H-7), 1.81 (2H, m, H-8). ^13^C-NMR (CD_3_OD, 75 MHz): δ 138.3 (C-1), 114.1 (C-2), 152.0 (C-3), 146.8 (C-4), 119.5 (C-5), 122.0 (C-6), 32.8 (C-7), 35.5 (C-8), 62.2 (C-9), 56.5 (OCH_3_), 62.0 (C-1′, C-3′), 83.4 (C-2′). Based on the above results and compared the spectral data with literature [20], compound 2 was identified as 4-*O*-(2-Hydroxy-1-hydroxymethylethyl)-dihydroconiferyl alcohol.

*Oleic acid* (3). Yellow syrup; Molecular weight of 282 and molecular formula of C_18_H_34_O_2_. ^1^H-NMR (CD_3_OD, 500 MHz): δ 5.27-5.39 (2H, m, H-9 and H-10), 2.26 (2H, t, *J* = 7.4 Hz, H-2), 2.05 (4H, q-*like*, *J* = 6.8 Hz, H-8 and H-11), 1.58 (2H, m, H-3), 1.23-1.39 (20H, m, H-4–H-7 and H-12–H-17), 0.90 (3H, t, *J* = 7.0 Hz, H-18). ^13^C-NMR (CD_3_OD, 125 MHz): δ 177.7 (s, C-1), 130.9 (d, C-9), 129.1 (d, C-10), 35.0 (t, C-2), 33.1 (t, C-16), 30.2-30.8 (t, C-4–C-7 and C-12–C-15), 28.1 (t, C-8 and C-11), 26.1 (t, C-3), 23.6 (t, C-17), 14.4 (q, C-18).

*Palmitic acid* (4). Colorless glue-like solid; Molecular weight of 256 and molecular formula of C_16_H_32_O_2_. ^1^H-NMR (CDCl_3_, 500 MHz): δ 2.33 (2H, t, *J* = 7.4 Hz, H-2), 1.62 (2H, m, H-3), 1.16-1.37 (24H, m, H-4–H-15), 0.87 (3H, t, *J* = 7.1 Hz, H-16). ^13^C-NMR (CDCl_3_, 125 MHz): δ 179.6 (s, C-1), 34.0 (t, C-2), 31.9 (t, C-14), 29.0-29.7 (t, C-4–C-13), 24.7 (t, C-3), 22.6 (t, C-15), 14.1 (q, C-16).

*desmethylisoophiopogonone B* [*5*,*7*-*dihydroxy*-*3*-(*4*′-*hydroxybenzyl*)-*8*- *methylchromone*] (5). Pale yellow needle-like crystals; Its positive-ion ESI-MS (*m*/*z*) displayed quasi-molecular ion peaks at 299 [M + H]^+^, indicating a molecular weight of 298, molecular formula of C_17_H_14_O_5_. ^1^H-NMR (CD_3_OD, 300 MHz): δ 7.72 (1H, s, H-2), 7.09 (2H, d, *J* = 8.4 Hz, H-2′, H-6′), 6.71 (2H, d, *J* = 8.4 Hz, H-3′, H-5′), 6.21 (1H, s, H-6), 3.59 (2H, s, H-9), 2.08 (3H, s, 8-CH_3_). ^13^C-NMR (CD_3_OD, 75 MHz): δ 155.3 (C-2), 123.8 (C-3), 183.2 (C-4), 160.8 (C-5), 99.1 (C-6), 163.3 (C-7), 103.5 (C-8), 30.7 (C-9), 157.2 (C-10), 105.9 (C-11), 130.8 (C-1′), 130.9 (C-2′, C-6′), 116.3 (C-3′, C-5′), 157.0 (C-4′), 7.3 (8-CH_3_). Based on the above results and compared the spectral data with literature [21], compound 5 was identified as 5,7-dihydroxy-3-(4′-hydroxybenzyl)-8- methylchromone.

*5*,*7*-*dihydroxy*-*6*-*methyl*-*3*-(*4*’-*hydroxy*-*benzyl*)-*chroman*-*4*-*one* (6). Yellow amorphous powder; Its positive-ion ESI-MS (*m*/*z*) displayed quasi-molecular ion peaks at 323 [M + Na]^+^, indicating a molecular weight of 300, molecular formula of C_17_H_16_O_5_. ^1^H-NMR (CD_3_OD, 300 MHz): δ 7.03 (2H, d, *J* = 8.4 Hz, H-2′, H-6′), 6.72 (2H, d, *J* = 8.4 Hz, H-3′, H-5′), 5.87 (1H, s, H-8), 4.18 (1H, dd, *J* = 11.4, 4.1 Hz, H-2a), 4.01 (1H, dd, *J* = 11.4, 7.0 Hz, H-2b), 3.07 (1H, dd, *J* = 13.5, 4.2 Hz, H-9a), 2.74 (1H, m, H-3), 2.60 (1H, dd, *J* = 13.5, 10.4 Hz, H-9b), 1.92 (3H, s, 6-CH_3_). ^13^C-NMR (CD_3_OD, 75 MHz): δ 70.0 (C-2), 48.2 (C-3), 199.5 (C-4), 162.9 (C-5), 105.3 (C-6), 166.0 (C-7), 94.9 (C-8), 33.1 (C-9), 162.2 (C-10), 102.6 (C-11), 130.3 (C-1′), 131.1 (C-2′, C-6′), 116.4 (C-3′, C-5′), 157.1 (C-4′), 7.0 (6-CH_3_). Based on the above results and compared the spectral data with literature [22], compound 6 was identified as 5,7-dihydroxy-6-methyl-3-(4′-hydroxy-benzyl)-chroman-4-one.

*5*,*7*-*dihydroxy*-*6*-*methyl*-*3*-(*4*'-*hydroxybenzyl*) *chromone* (7). White amorphous powder; Its positive-ion ESI-MS (*m*/*z*) displayed quasi-molecular ion peaks at 299 [M + H]^+^, indicating a molecular weight of 298, molecular formula of C_17_H_14_O_5_. ^1^H-NMR (CD_3_OD, 300 MHz): δ 7.63 (1H, s, H-2), 7.08 (2H, d, *J* = 8.4 Hz, H-2′, H-6′), 6.71 (2H, d, *J* = 8.4 Hz, H-3′, H-5′), 6.30 (1H, s, H-8), 3.59 (2H, s, H-9), 2.02 (3H, s, 6-CH_3_). ^13^C-NMR (CD_3_OD, 75 MHz): δ 154.9 (C-2), 124.2 (C-3), 182.8 (C-4), 160.2 (C-5), 108.7 (C-6), 163.8 (C-7), 93.6 (C-8), 30.8 (C-9), 157.6 (C-10), 105.6 (C-11), 130.8 (C-1′), 130.9 (C-2′, C-6′), 116.3 (C-3′, C-5′), 157.0 (C-4′), 7.4 (6-CH_3_). Based on the above results and compared the spectral data with literature [22], compound 7 was identified as 5,7-dihydroxy-6-methyl-3-(4′-hydroxybenzyl) chromone.

*3*-(*2*,*4*-*Dihydroxybenzyl*)-*5*-*hydroxy*-*7*,*8*-*dimethoxy*-*6*-*methylchroman*-*4*-*one* (8). Yellow syrup; Its positive-ion ESI-MS (*m*/*z*) displayed quasi-molecular ion peaks at 361 [M + H]^+^, indicating a molecular weight of 360, molecular formula of C_19_H_20_O_7_. ^1^H-NMR (CD_3_OD, 700 MHz): δ 6.86 (1H, d, *J* = 8.1 Hz, H-6′), 6.29 (1H, d, *J* = 2.2 Hz, H-3′), 6.23 (1H, dd, *J* = 8.1, 2.2 Hz, H-5′), 4.30 (1H, dd, *J* = 11.4, 4.1 Hz, H-2a), 4.16 (1H, dd, *J* = 11.4, 7.6 Hz,H-2b), 3.92 (3H, s, 7-OCH_3_), 3.72 (3H, s, 8-OCH_3_), 3.14 (1H, dd, *J* = 13.5, 5.0 Hz, H-9a), 2.98 (1H, m, H-3), 2.56 (1H, dd, *J* = 13.5, 9.8 Hz, H-9b), 1.97 (3H, s, 6-CH_3_). ^13^C-NMR (CD_3_OD, 75 MHz): δ 70.9 (C-2), 46.8 (C-3), 201.3 (C-4), 157.8 (C-5), 111.2 (C-6), 160.9 (C-7), 134.2 (C-8), 28.1 (C-9), 154.2 (C-10), 105.2 (C-11), 116.6 (C-1′), 157.6 (C-2′), 103.5 (C-3′), 158.3 (C-4′), 107.4 (C-5′), 132.6 (C-6′), 7.7 (6-CH_3_), 61.3 (7-OCH_3_), 61.7 (8-OCH_3_). Based on the above results and compared the spectral data with literature [21], compound 8 was identified as 3-(2,4-Dihydroxybenzyl)-5-hydroxy-7,8-dimethoxy-6-methylchroman-4- one.

*5*,*7*-*dihydroxy*-*3*-(*4*' - *hydroxybenzyl*) *chromone* (9). Yellow needle-like crystals; Its positive-ion ESI-MS (*m*/*z*) displayed quasi-molecular ion peaks at 285 [M + H]^+^, indicating a molecular weight of 284, molecular formula of C_16_H_12_O_5_. ^1^H-NMR (CD_3_OD, 500 MHz): δ 7.68 (1H, s, H-2), 7.09 (2H, d, *J* = 8.6 Hz, H-2′, H-6′), 6.71 (2H, d, *J* = 8.6 Hz, H-3′, H-5′), 6.26 (1H, d, *J* = 2.1Hz, H-8), 6.16 (1H, d, *J* = 2.1 Hz, H-6), 3.60 (2H, s, H-9). ^13^C-NMR (CD_3_OD, 125 MHz): δ 155.2 (C-2), 124.4 (C-3), 182.8 (C-4), 163.5 (C-5), 99.9 (C-6), 165.9 (C-7), 94.7 (C-8), 30.7 (C-9), 159.9 (C-10), 106.0 (C-11), 130.7 (C-1′), 130.9 (C-2′, C-6′), 116.3 (C-3′, C-5′), 157.0 (C-4′). Based on the above results and compared the spectral data with literature [23], compound 9 was identified as 5,7-dihydroxy-3-(4' - hydroxybenzyl) chromone

*4*'-*O*-*Demethylophiopogonanone E* (10) was obtained as a white amorphous powder with molecular formula of C_18_H_18_O_7_ determined by its HR-ESI-MS at m/z 347.1160 [M + H]^+^ (calcd. for 347.1131) and the NMR spectra. ^1^H-NMR (CD_3_OD, 500 MHz): δ 6.86 (1H, d, *J* = 8.1 Hz, H-6′), 6.29 (1H, d, *J* = 2.4 Hz, H-3′), 6.23 (1H, dd, *J* = 8.1, 2.4 Hz, H-5′), 4.27 (1H, dd, *J* = 11.3, 4.6 Hz, H-2a), 4.14 (1H, dd, *J* = 11.3, 7.4 Hz, H-2b), 3.71 (3H, s, OCH_3_), 3.14 (1H, dd, *J* = 13.7, 4.9 Hz, H-9a), 2.93 (1H, m, H-3), 2.57 (1H, dd, *J* = 13.7, 10.0 Hz, H-9b), 1.95 (3H, s, 6-CH_3_). ^13^C-NMR (CD_3_OD, 125 MHz): δ 70.8 (C-2), 46.6 (C-3), 200.4 (C-4), 158.3 (C-5), 105.1 (C-6), 158.8 (C-7), 129.0 (C-8), 28.3 (C-9), 153.1 (C-10), 102.4 (C-11), 116.8 (C-1′), 157.6 (C-2′), 103.5 (C-3′), 158.5 (C-4′), 107.5 (C-5′), 132.6 (C-6′), 7.2 (6-CH_3_), 61.6 (OCH_3_). The ^1^H NMR spectrum of 10 showed the presence of a methoxy group at δ 3.71 (3H, s), a methyl group attached to an aromatic nucleus at δ 1.95 (3H, s, 6-CH_3_), and three ABX aromatic proton signals appeared at δ 6.29 (1H, d, *J* = 2.4 Hz, H-3′), 6.23 (1H, dd, *J* = 8.1, 2.4 Hz, H-5′), and 6.86 (1H, d, *J* = 8.1 Hz, H-6′). In addition, the ^1^H NMR signals at δ 4.27 (1H, dd, *J* = 11.3, 4.6 Hz, H-2a), 4.14 (1H, dd, *J* = 11.3, 7.4 Hz, H-2b), and 2.93 (1H, m, H-3) were observed, which indicated the γ-dihydropyrone moiety. Combined with the two benzylmethylene protons appeared at δ_H_ 3.14 (1H, dd, *J* = 13.7, 4.9 Hz, H-9a) and 2.57 (1H, dd, *J* = 13.7, 10.0 Hz, H-9b), compound 10 was presumed to be a homoisoflavonoid derivative [19]. After carefully analysis of the NMR spectra of 10, the spectral properties were found very similar to those of ophiopogonanone E [19]. The notable difference was the presence of a hydroxy group instead of a methoxy group at C-4′ in 10, compared with ophiopogonanone E. The change and the structure of 10 were further confirmed by HMBC correlations from δ_H_ 3.71 (3H, s, OCH_3_) to C-8 (δ_C_ 129.0), from δ_H_ 1.95 (3H, s, 6-CH_3_) to C-6 (δ_C_ 105.1), C-5 (δ_C_ 158.3) and C-7 (δ_C_ 158.8), from δ_H_ 4.27 (1H, dd, *J* = 11.3, 4.6 Hz, H-2a), 4.14 (1H, dd, *J* = 11.3, 7.4 Hz, H-2b), 3.14 (1H, dd, *J* = 13.7, 4.9 Hz, H-9a), and 2.57 (1H, dd, *J* = 13.7, 10.0 Hz, H-9b) to C-4 (δ_C_ 200.4), and from δ_H_ 6.86 (1H, d, *J* = 8.1 Hz, H-6′) to C-9 (δ_C_ 28.3), C-2′ (δ_C_ 157.6), and C-4′ (δ_C_ 158.5). Therefore, the chemical structure of 10 was determined to be 3-(2,4-dihydroxybenzyl)-5,7-dihydroxy-8- methoxy-6-methylchroman-4-one and named as 4′-*O*-demethylophiopogonanone E. There have been no related reports about the structure of compound 10 in SciFinder scholar, suggesting that the compound may be a new compound, the NMR signals of this homoisoflavonoid were reported and completely assigned for the first time.

*Ophiopogonone D* (11). Yellow amorphous powder; Its positive-ion ESI-MS (*m*/*z*) displayed quasi-molecular ion peaks at 315 [M + H]^+^, 651 [2 M + Na]^+^, indicating a molecular weight of 314, molecular formula of C_17_H_14_O_6_. ^1^H-NMR (CD_3_OD, 500 MHz): δ 7.65 (1H, s, H-2), 6.96 (1H, d, *J* = 8.2 Hz, H-6′), 6.31 (1H, s, H-8), 6.30 (1H, d, *J* = 2.5Hz, H-3′), 6.25 (1H, dd, *J* = 8.2, 2.4Hz, H-5′), 3.57 (2H, s, H-9), 2.03 (3H, s, H-12). ^13^C-NMR (CD_3_OD, 125 MHz): δ 155.1 (C-2), 123.4 (C-3), 183.2 (C-4), 160.1 (C-5), 108.8 (C-6), 164.0 (C-7), 93.7 (C-8), 25.6 (C-9), 157.7 (C-10), 105.5 (C-11), 7.4 (C-12), 117.2 (C-1′), 158.4 (C-2′), 103.9 (C-3′), 157.2 (C-4′), 107.9 (C-5′), 132.3 (C-6′). Based on the above results and compared the spectral data with literature [24], compound 11 was identified as Ophiopogonone D.

*5*,*7*-*dihydroxy*-*6*-*methyl*-*3*-(*2*',*4*'-*dihydroxybenzyl*) *chroman*-*4*-*one* (12). Yellow needle-like crystals; Molecular weight of 316, molecular formula of C_17_H_16_O_6_. ^1^H-NMR (CD_3_OD, 500 MHz): δ 6.86 (1H, d, *J* = 8.2 Hz, H-6′), 6.30 (1H, d, *J* = 2.4 Hz, H-3′), 6.24 (1H, dd, *J* = 8.2, 2.4 Hz, H-5′), 5.88 (1H, s, H-8), 4.20 (1H, dd, *J* = 11.4, 4.2 Hz, H-2a), 4.05 (1H, dd, *J* = 11.4, 7.6 Hz, H-2b), 3.15 (1H, dd, *J* = 13.7, 4.8 Hz, H-9a), 2.93 (1H, m, H-3), 2.55 (1H, dd, *J* = 13.7, 10.1 Hz, H-9b), 1.93 (3H, s, 6-CH_3_). ^13^C-NMR (CD_3_OD, 125 MHz): δ 70.6 (C-2), 46.5 (C-3), 200.3 (C-4), 165.9 (C-5), 105.2 (C-6), 162.8 (C-7), 94.8 (C-8), 28.2 (C-9), 162.3 (C-10), 102.6 (C-11), 116.9 (C-1′), 157.5 (C-2′), 103.5 (C-3′), 158.3 (C-4′), 107.5 (C-5′), 132.6 (C-6′), 7.0 (6-CH_3_). Based on the above results and compared the spectral data with literature [25], compound 12 was identified as 5,7-dihydroxy-6-methyl-3-(2′,4′- dihydroxybenzyl) chroman-4-one.

*Daucosterol* (13). White powder; Molecular weight of 576, molecular formula of C_35_H_60_O_6_. ^1^H-NMR (DMSO-*d*_6_, 500 MHz): δ 5.32 (1H, br s, H-6), 4.84-4.93 (3H, 2′-OH, 3′-OH, 4′-OH), 4.44 (1H, t, *J* = 5.5 Hz, 6′-OH), 4.21 (1H, d, *J* = 7.8 Hz, H-1′), 2.35 (1H, br d, *J* = 12.4 Hz, H-4a), 2.11 (1H, br t, *J* = 12.4 Hz, H-4b), 0.94 (3H, s, H-19), 0.89 (3H, d, *J* = 6.3 Hz, H-21), 0.81 (3H, t, *J* = 6.6 Hz, H-29), 0.80 (3H, d, *J* = 6.9 Hz, H-27), 0.78 (3H, d, *J* = 6.9 Hz, H-26), 0.64 (3H, s, H-18). ^13^C-NMR (DMSO-*d*_6_, 125 MHz): δ 36.8 (C-1), 29.3 (C-2), 76.9 (C-3), 38.3 (C-4), 140.5 (C-5), 121.2 (C-6), 31.4 (C-7, C-8), 49.6 (C-9), 36.2 (C-10), 20.6 (C-11), 40.1 (C-12), 41.9 (C-13), 55.4 (C-14), 23.9 (C-15), 27.8 (C-16), 56.2 (C-17), 11.7 (C-18), 19.1 (C-19), 35.5 (C-20), 18.6 (C-21), 33.4 (C-22), 25.4 (C-23), 45.1 (C-24), 28.7 (C-25), 18.9 (C-26), 19.7 (C-27), 22.6 (C-28), 11.8 (C-29), 100.8 (C-1'), 73.5 (C-2'), 76.8 (C-3'), 70.1 (C-4'), 76.8 (C-5'), 61.1 (C-6'). Based on the above results and compared the spectral data with literature [26], compound 13 was identified as Daucosterol.

### Effects of isolated compounds (1–13) on Cell Viability of RAW 264.7Macrophages

**Fig. 3:**
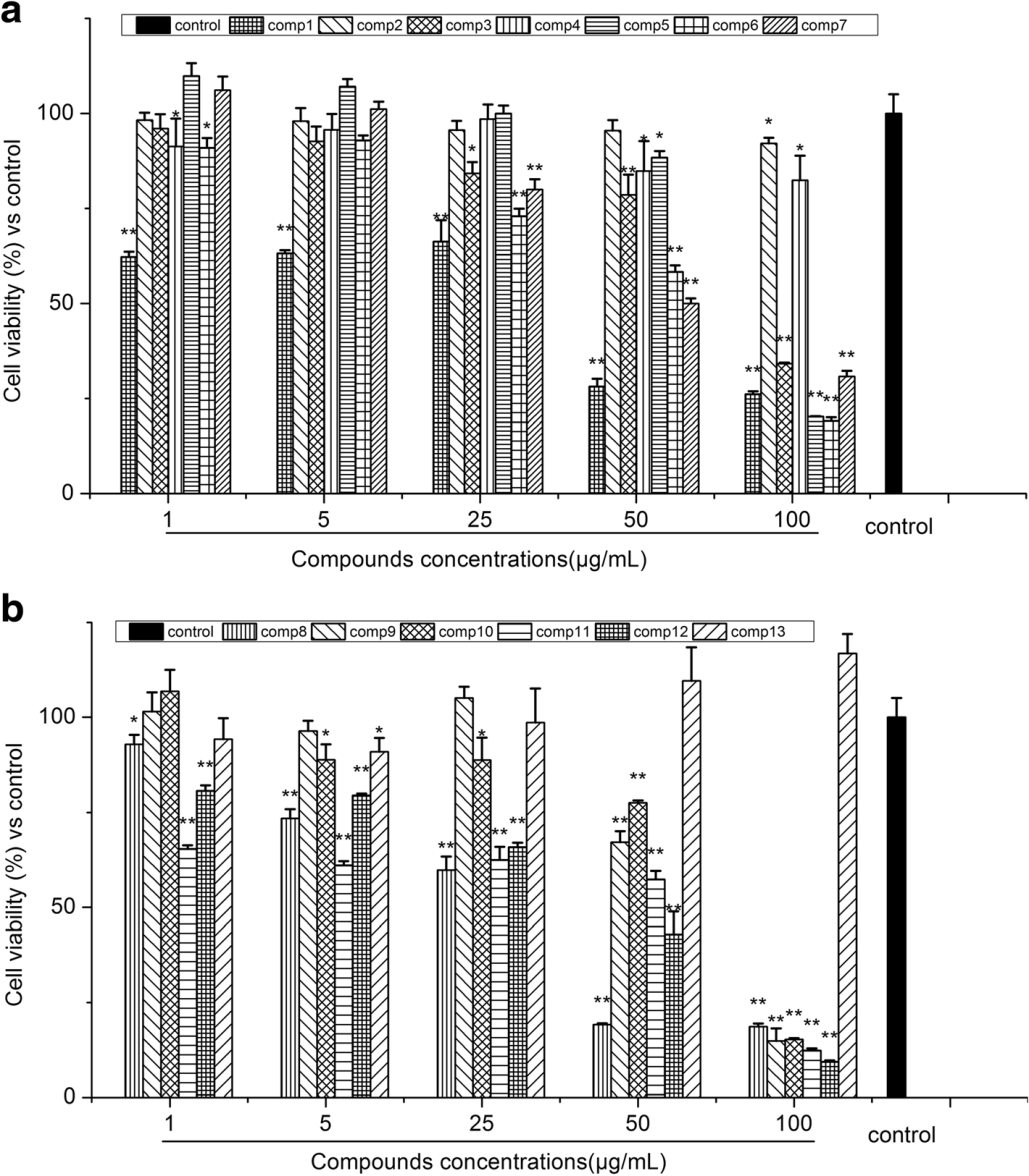
Effects of isolated compounds 1–7 (**a**) and 8–13 (**b**) on cell viability of RAW 264.7 macrophages. Cells were incubated for 24 h with the indicated concentrations of compounds (1, 5, 25, 50 and 100 μg/mL). data are expressed as mean ± S.D. Of three independent experiments. * *p* < 0.05 and ** *p* < 0.01 versus the control group

The cytotoxicity of these compounds on the proliferation of RAW264.7 cells was measured using the 3-(4,5-dimethylthiazol-2-yl)-2,5-diphenyltetrazolium-bromide (MTT) assay [27]. The results were shown as relative cell viability referred to control (equal to 100%). In this study, 13 compounds of *O. japonicas* were shown different cytotoxicity effect to RAW 264.7 cells. The safe concentration of compound 2, 4 and 13 is 100 μg/mL, while compound 3, 5 and 10 is 50 μg/mL, which is the cell survival probability more than 80%, the other compounds have some cytotoxicity at the concentrations (1–100 μg/mL) (Fig. 3).

### Effects of isolated compounds (1–13) on the Production of NO in LPS-induced RAW264.7 Macrophages

**Fig. 4:**
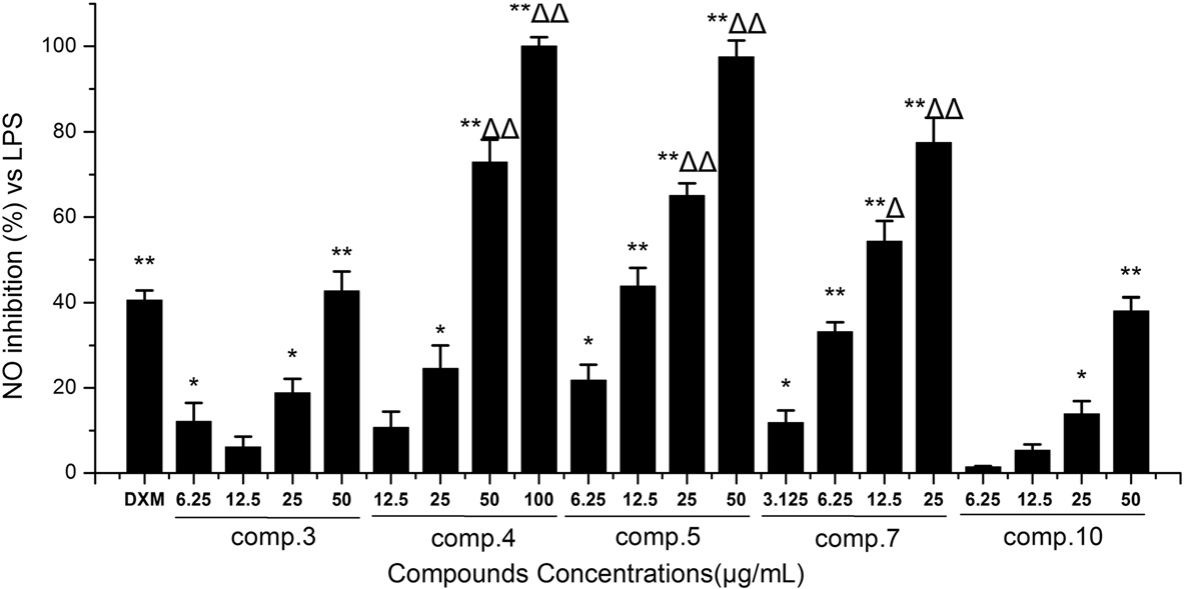
Effects of isolated compounds (3–5, 7 and 10) on the production of NO in LPS-induced RAW264.7 macrophages. Cells were pre-treated for 2 h with indicated concentrations of (3–5, 7 and 10) and then stimulated with LPS (final concentration 1 μg/mL) for another 24 h. Data are expressed as mean ± S.D. Of three independent experiments. * *p* < 0.05 and ** *p* < 0.01 versus the LPS group, △*p* < 0.05 and △△*p* < 0.01 versus the DXM group

To evaluate the effect of compounds (1–13) on NO production in LPS-stimulated RAW 264.7 cells, we measured the nitrite concentration in the cultured media using Griess reagent [28]. Compared with the LPS group, the anti-inflammatory activities of compounds 3, 4, 5, 7 and 10 increased in accordance with the increase in concentration. As shown in Fig. 4, compounds 3, 4, 5, 7 and 10 displayed an inhibitory potency, with IC_50_ values of 80.2 ± 2.3, 33.4 ± 2.9, 14.1 ± 1.5, 10.9 ± 0.8 and 66.4 ± 3.5 μg/mL, respectively. Moreover, compounds 4, 5 and 7 showed stronger anti-inflammatory abilities than the positive control DXM at the concentration of 50 μg/mL (*p* < 0.01), in which compounds 5 and 7 belonged to homoisoflavonoids. Thus, it is possible to demonstrate that the anti-inflammatory activity of *O. japonicas* might be derived from its rich in homoisoflavonoids compounds, however, there are few literatures reported on its anti-inflammatory mechanism.

### Effects of the compounds 3–5, 7 and 10 on the Production of Pro-Inflammatory Cytokines (IL-1β and IL-6) in LPS-induced RAW264.7 Macrophages

**Fig. 5:**
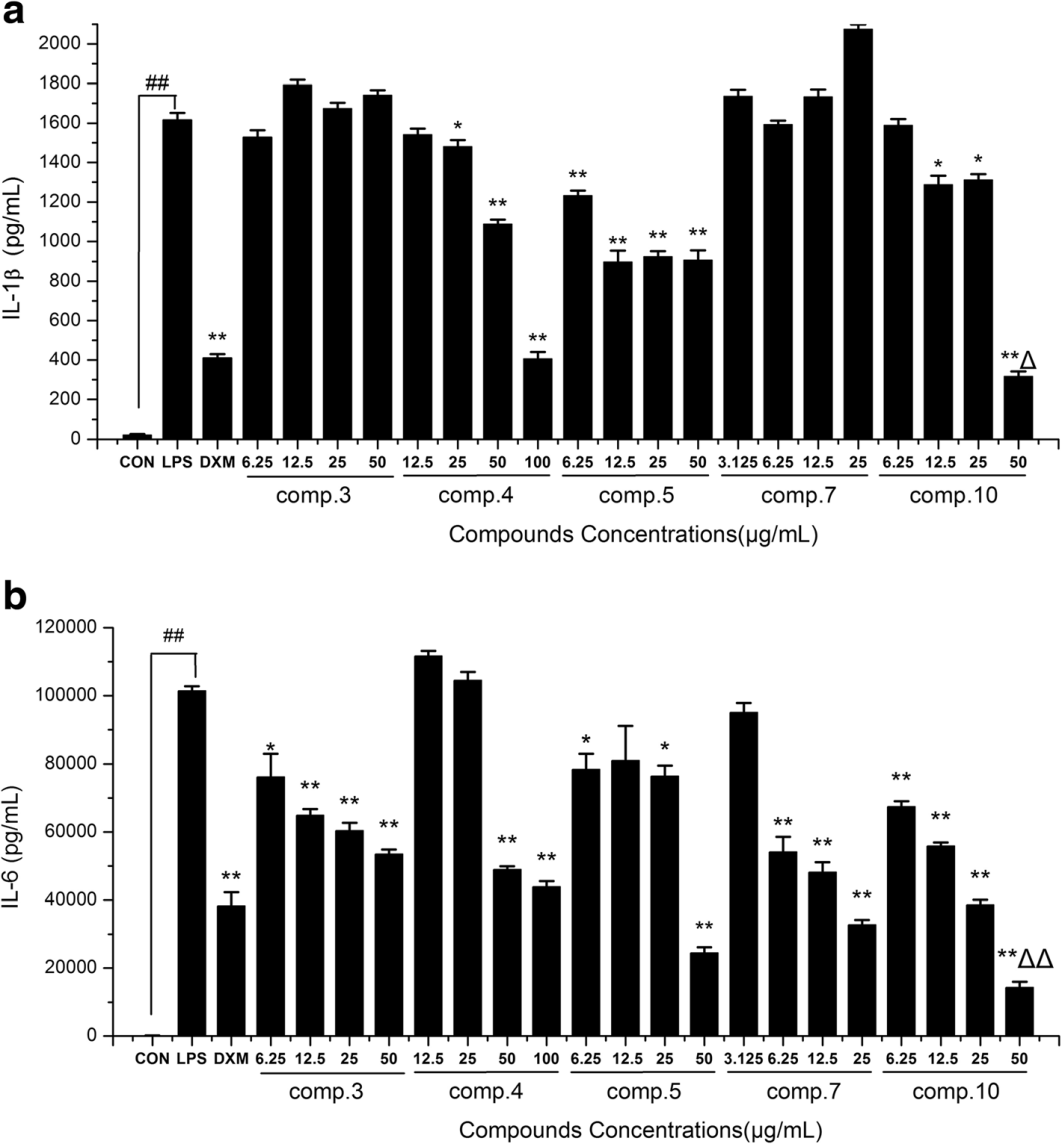
Effects of the compounds 3–5, 7 and 10 on the production of pro-inflammatory cytokines IL-1β (**a**) and IL-6 (**b**) in LPS-induced RAW264.7 macrophages. Cells were pre-treated for 2 h with indicated concentrations of 3–5, 7 and 10 and then stimulated with LPS (final concentration 1 μg/mL) for another 24 h. Data are expressed as mean ± S.D. Of three independent experiments. ## *p* < 0.01 as compared with the control group, * *p* < 0.05 and ** *p* < 0.01 versus the LPS group, △*p* < 0.05 and △△*p* < 0.01 versus the DXM group

Because IL-1β and IL-6 are early secreted pro-inflammatory cytokines and their elevated levels can be detected in a variety of acute and chronic inflammatory diseases [29], we measured IL-1β and IL-6 production in the supernatant of RAW 264.7 cells by ELISA. The LPS group significantly increased IL-1β and IL-6 production compared with the control group. The release of both cytokines were remarkably decreased in a dose-dependent manner (*p* < 0.05) in the groups pretreated with compounds 3, 4, 5, 7 and 10 (Fig. 5a and b). Moreover, the IL-1β IC_50_ values of compounds 4, 5 and 10 were 65.3 ± 6.8, 64.3 ± 7.9 and 32.5 ± 3.5 μg/mL, respectively. In addition to IL-6, compounds 3, 4, 5, 7 and 10 displayed an inhibitory potency, with IC_50_ values of 58.9 ± 6.8, 71.6 ± 11.7, 32.4 ± 3.6, 11.5 ± 2.8 and 13.4 ± 2.3 μg/mL, respectively. This result indicated that 4'-*O*-Demethylophiopogonanone E (10) efficiently suppressed LPS-induced IL-1β and IL-6 production, especially, it showed stronger anti-inflammatory ability than DXM at the concentration of 50 μg/mL (*p* < 0.05). Its effect on the LPS-induced IL-1β, IL-6 and iNOS mRNA expressions needs further investigation.

### Effects of 4'-*O*-Demethylophiopogonanone E (10) on the mRNA Expression of IL-1β, IL-6 and iNOS in LPS-induced RAW264.7 Macrophages

**Fig. 6:**
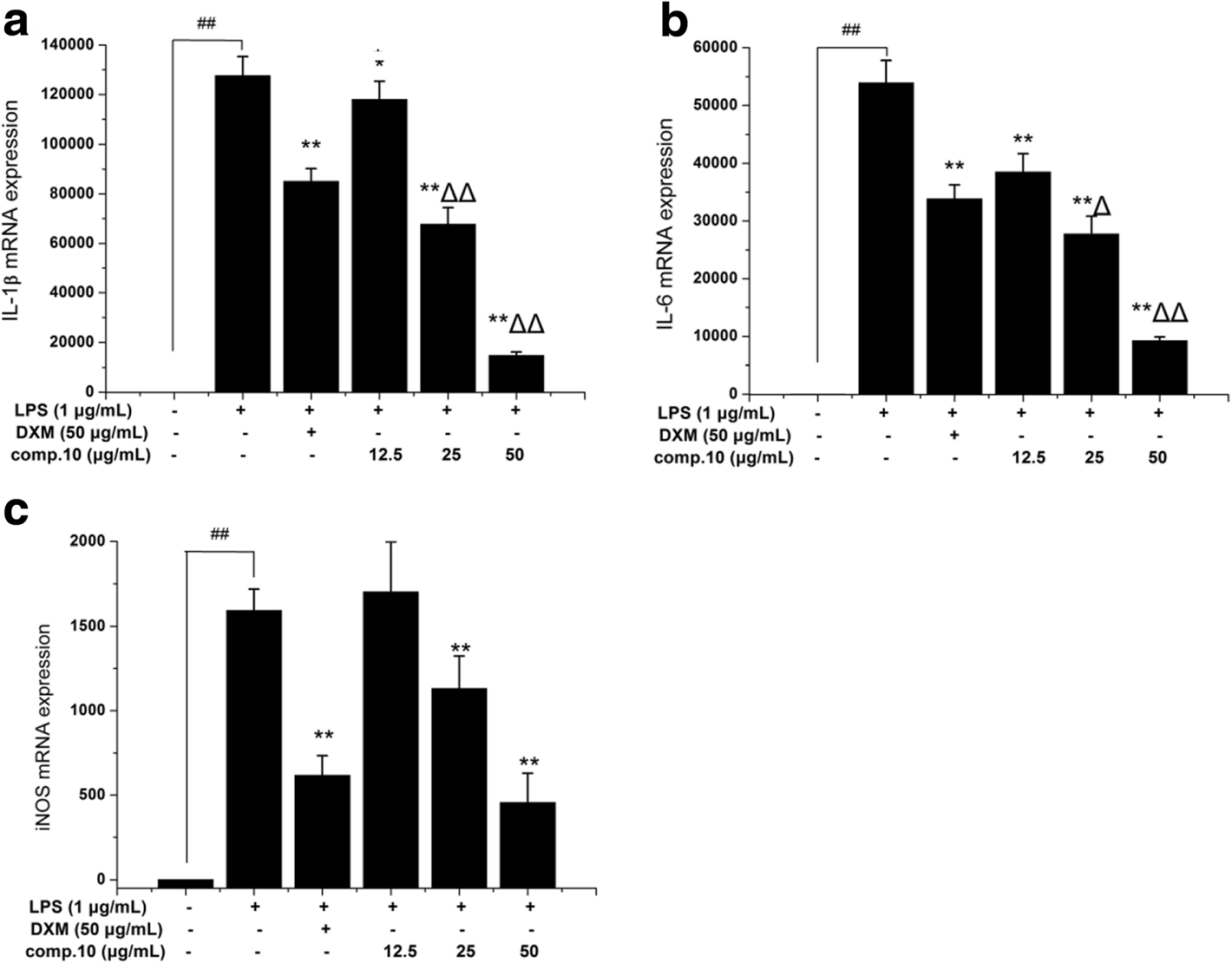
Effects of 4'-*O*-demethylophiopogonanone E (10) on the mRNA expression of IL-1β (**a**), IL-6 (**b**) and iNOS (**c**) in LPS-induced RAW264.7 macrophages. Cells were pre-treated for 2 h with indicated concentrations of **10** and then stimulated with LPS (final concentration 1 μg/mL) for another 12 h. Data are expressed as mean ± S.D. Of three independent experiments. ## *p* < 0.01 as compared with the control group, * *p* < 0.05 and ** *p* < 0.01 versus the LPS group, △ *p* < 0.05 and △△ *p* < 0.01 versus the DXM group

4'-*O*-Demethylophiopogonanone E (10) exhibited strong inhibitory effect on the LPS-induced IL-1β and IL-6 production, however, few studies were involved in the exact mechanism of the anti-inflammatory effect of 4'-*O*-Demethylophiopogonanone E. As shown in Fig. 6, compared to the control group, treatment with LPS for 12 h significantly (*p* < 0.01) increased mRNA expression of IL-1β, IL-6 and iNOS. Additionally, pre-treatment with 4'-*O*-Demethylophiopogonanone E (at the concentration of 25, 50 μg/mL) for 2 h could significantly inhibit the expression of IL-1β, IL-6 and iNOS in a dose-dependent manner (*p* < 0.01). The results suggested that 4'-*O*-Demethylophiopogonanone E decreased NO, IL-1β and IL-6 production by decreasing the iNOS, IL-1β and IL-6 mRNA expression at the transcriptional level.

### Effects of 4'-*O*-Demethylophiopogonanone E (10) on MAPKs Signaling Pathways Activation in LPS-induced RAW264.7 Macrophages

**Fig. 7:**
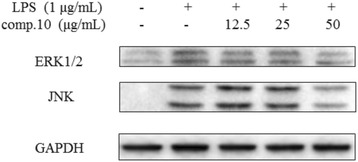
Effects of 4'-*O*-demethylophiopogonanone E (10) on phosphorylation of MAPKs activation in LPS-induced RAW 264.7 macrophages. Cells were pre-treated for 2 h with indicated concentrations of 10 and then stimulated with LPS (final concentration 1 μg/mL) for 1 h. The blots shown are representative of three blots yielding similar results

Previous studies have indicated that IL-1β and IL-6 are crucial factors involved in all kinds of inflammatory processes that can be regulated by activation of NF-κB [30–32]. In addition, MAPKs are also known to be important for the transcriptional NF-κB pathway activation and are related to iNOS expression [33]. To investigate whether the suppression of inflammatory responses by 4'-*O*-Demethylophiopogonanone E was mediated through the MAPKs pathways, we estimated the effects of compound 10 on the LPS-stimulated phosphorylation of ERK1/2 and JNK in RAW 264.7 cells by Western blotting. As shown in Fig. 7, this research demonstrated that LPS significantly increased the phosphorylation of MAPKs signaling proteins, while pre-treatment with compound 10 considerably inhibited the phosphorylation of ERK1/2 and JNK, however it did not have a distinct dose-dependent pattern. In conclusion, our findings suggested that 4'-*O*-Demethylophiopogonanone E might decrease NO, IL-1β and IL-6 production *via* inhibitions of the MAPKs signaling pathways.

## Conclusion

In this study, a new compound 4'-*O*-Demethylophiopogonanone E was isolated and identified from the rhizome of *O. japonicas*. Moreover, we firstly investigated the effect and mechanism of 4'-*O*-Demethylophiopogonanone E on LPS-stimulated NO and pro-inflammatory cytokines including IL-1β and IL-6. NO is synthesized from L-arginine by iNOS, and its overproduction is involved in cytotoxicity and tissue damage in the inflammatory process [34, 35]. Overexpression of iNOS by inflammatory agents accompanied with inflammatory disorder including IL-1β and IL-6, which are resulted in acute and chronic response to inflammatory diseases [36]. In the present study, we clearly demonstrated that 4'-*O*-Demethylophiopogonanone E significantly inhibited LPS-stimulated NO production and pro-inflammatory cytokines (IL-1β and IL-6) in macrophages *via* downregulation of the mRNA expressions of iNOS, IL-1β and IL-6 (Fig. 6).

Moreover, MAPKs have been involved in pro-inflammatory signaling cascades and large numbers of evidence has demonstrated that the activation of ERK1/2 and JNK is involved in up-regulation of nitric oxide and pro-inflammatory cytokines in LPS- induced macrophages [37, 38]. Thus, we assessed that 4'-*O*-Demethylophiopogonanone E inhibited the inflammatory response *via* blocking the phosphorylation of MAPKs in LPS-stimulated macrophages. In the results of 4'-*O*-Demethylophiopogonanone E pretreatment, we found that 4'-*O*-Demethylophiopogonanone E suppressed LPS-stimulated ERK1/2 and JNK MAPK phosphorylation (Fig. 7). This result suggests that 4'-*O*-Demethylophiopogonanone E exerted anti-inflammatory actions *via* inhibition of iNOS, IL-1β and IL-6 gene expressions through suppression of the MAPKs signaling pathways.

Consequently, based on these findings, we provide clear evidence and molecular basis for the anti-inflammatory mechanism of 4'-*O*-Demethylophiopogonanone E and show great potential as a novel herbal ingredient for the treatment of inflammatory diseases.
